# Photodynamic Therapy Efficacy of Novel Zinc Phthalocyanine Tetra Sodium 2-Mercaptoacetate Combined with Cannabidiol on Metastatic Melanoma

**DOI:** 10.3390/pharmaceutics14112418

**Published:** 2022-11-09

**Authors:** Nkune Williams Nkune, Gauta Gold Matlou, Heidi Abrahamse

**Affiliations:** Laser Research Centre, Faculty of Health Sciences, University of Johannesburg, P.O. Box 17011, Johannesburg 2028, South Africa

**Keywords:** photodynamic therapy, zinc phthalocyanine, cannabidiol, skin cancer cells

## Abstract

This work reports for the first time on the synthesis, characterization, and photodynamic therapy effect of a novel water-soluble zinc (II) 2(3), 9(10), 16(17), 23(24)-tetrakis-(sodium 2-mercaptoacetate) phthalocyanine (ZnPcTS41), on metastatic melanoma cells (A375) combined with cannabidiol (CBD). The ZnPcTS41 structure was confirmed using FTIR, NMR, MS, and elemental analysis while the electronic absorption spectrum was studied using UV-VIS. The study reports further on the dose-dependent effects of ZnPcTS41 (1–8 µM) and CBD alone (0.3–1.1 µM) at 636 nm with 10 J/cm^2^ on cellular morphology and viability. The IC_50_ concentrations of ZnPcTS41 and CBD were found to be 5.3 µM and 0.63 µM, respectively. The cytotoxicity effects of the ZnPcTS41 enhanced with CBD on A375 cells were assessed using MTT cell viability assay, ATP cellular proliferation and inverted light microscopy. Cell death induction was also determined via Annexin V-FITC-PI. The combination of CBD- and ZnPcTS41-mediated PDT resulted in a significant reduction in cell viability (15%***) and an increase in the late apoptotic cell population (25%*). These findings suggest that enhancing PDT with anticancer agents such as CBD could possibly obliterate cancer cells and inhibit tumor recurrence.

## 1. Introduction

Melanoma is the deadliest form of skin cancer with a rapidly increasing incidence and a grim prognosis for patients diagnosed in the advanced stages [1]. Despite several treatments available for treating this cancer through surgical excision, chemotherapy, radiation therapy and immunotherapy, resistance to therapies remains one of the major challenges, especially if the tumor has metastasized [2]. In addition, the above-mentioned treatments cause adverse side effects on the normal tissues and therefore it is imperative to develop new agents that are more effective and less toxic to patients [3]. Photodynamic therapy (PDT) is a promising modality for the treatment of cancer because it is non-invasive and selectively damages the cancerous tissue, minimizing damage to adjacent healthy structures [4]. PDT involves three fundamentals: a photosensitive agent, molecular oxygen, and visible light [5]. The photoactivation of photosensitizers (PSs) during the PDT procedure triggers photochemical reactions that lead to the production of tumoricidal reactive oxygen species (ROS), which ultimately annihilate cancer cells [6,7,8].

Melanoma is the most aggressive skin cancer and so metastasis of this disease is normally eminent [2]. Once the cancer has disseminated to other parts of the body, it is referred to as metastatic melanoma (MM) or stage IV melanoma [3]. Researchers have identified stem-like cells in MM, termed melanoma stem-like cells (MSCs), that are responsible for tumorigenesis, drug resistance, and metastasis [9,10]. Studies reported that MSCs are involved in the resistance to PDT, metastatic process, and lead to secondary recurrence of MM [10]. Furthermore, another limitation of PDT treatment is the presence of melanin, which renders MM cells less susceptible to photodamage. Melanin acts as a filter that strongly absorbs light at wavelengths crucial for PDT (400–750 nm), preventing light penetration and excitation of the PS retained in tumor tissues [11]. Therefore, there is an urgent need to combine PDT with anticancer agents that could possibly eradicate primary tumor growth and inhibit secondary tumor growth by inducing anticancer immune responses that could control metastasis [12]. In this study, a novel water-soluble zinc phthalocyanine derivative is applied as a PS agent during in vitro PDT treatment of melanoma skin cancer (A375) and combined with anticancer agents (cannabidiol) to completely eradicate the primary and secondary recurrence of metastatic melanoma.

**Scheme 1:**
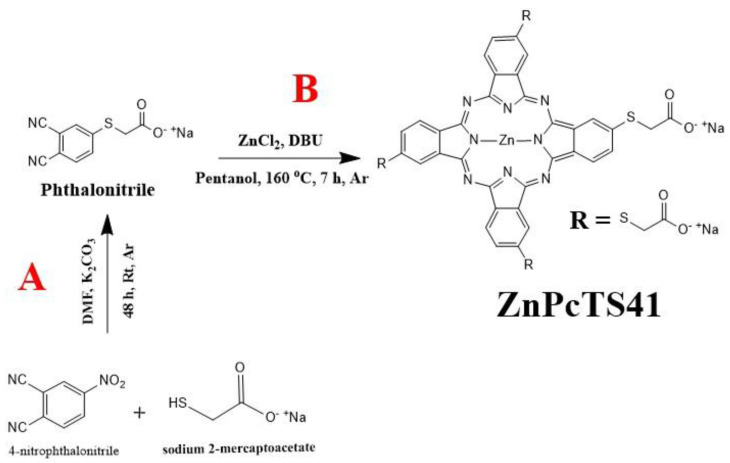
Schematic diagram illustrating the synthesis of (**A**) sodium 2-((2,3-dicyanophenyl)thiol)acetate (precursor phthalonitrile) and (**B**) synthesis of zinc (II) 2(3), 9(10), 16(17), 23(24)-tetrakis-(sodium 2-mercaptoacetate) phthalocyanines (ZnPcTS41).

Zinc phthalocyanines (ZnPc) are well known PS agents in PDT due to their ability to absorb light and transition through various photochemical and photophysical pathways to generate high yields of cytotoxic singlet oxygen and other reactive oxygen species [13,14,15]. This is the driving reason behind the synthesis and use of a novel ZnPc derivative in this study. Scheme 1. Phthalocyanines have excellent physicochemical properties that are improved by their target substituents of the benzene rings and the central metal [16,17,18,19]. Heavy metals such as zinc are inserted in the Pc macrocyclic structure with intention to accelerate intersystem crossing of excited molecules to the excited triple state through heavy atom effects [20,21], which promotes the generation of high yields of cytotoxic singlet oxygen. In addition, improved cellular uptake with negligible cytotoxicity and skin hypersensitivity made them popular in PDT applications [22]. More importantly, their strong absorption within the therapeutic window can facilitate maximum light penetration depth for the treatment of deep-seated tumors [22].

The phthalocyanine structure also allows for fine tuning by addition of different substituents on the peripheral (β) and non-peripheral (α) positions of the isoindole subunits (benzene rings) [23,24,25,26]. In this work, sodium 2-mercaptoacetate is used as a substituent on the peripheral position of the benzene rings of the macrocyclic Pc structure. The salt form improves water solubility of the complex and makes it easy to apply in PDT and subcellular localization studies [22]. The thiol group (sulfur) of the PC structure can further improve their water solubility and prevent them from aggregating, without significantly hampering their photophysical properties [27].

Cannabidiol is a non-psychoactive compound extracted from *Cannabis sativa*, which has attracted tremendous attention since it is a chemo-preventive and its pro-apoptotic effects counteract tumor neovascularization, cancer migration, adhesion, metastasizing, and possibly inhibit tumor relapse in patients [28]. Studies by Burch et al, 2021 [29] reported that CBD demonstrated a significant inhibitory effect on both in vivo- and in vitro-cultured melanoma cells [29]. CBD is used in this study to enhance the efficacy of PDT on melanoma skin cancer cells and improve the overall therapy by completely eradicating the recurrence of metastatic melanoma.

This work reports on the successful synthesis, characterization, and application of a novel water soluble ZnPc derivative (Scheme 1, labelled ZnPcTS41) as a PS agent when alone and when combined with CBD on skin cancer cells. To the best of our knowledge, this is the first time that sodium 2-mercaptoacetate is used as a substituent on Pc core structure. CBD on its own has no phototoxicity but has been demonstrated previously to be an effective in tumor growth destruction. The use of CBD in this work is to enhance the efficacy of the treatment and result in the total destruction of cancer cells.

## 2. Materials and Methods

### 2.1. Materials

Zinc chloride, aluminum chloride, dimethyl sulfoxide (DMSO) and dimethyl formamide (DMF), 1,3-diphenylisobenzofuran (DPBF), sodium 2-mercaptoacetate and 1-pentanol were purchased from Sigma-Aldrich. Tetrahydrofuran (THF), Methanol, Dichloromethane and 1,8-diazabicyclo [5.4.0] undec-7-ene (DBU) was also purchased from Sigma-Aldrich. Milipore water was obtained from the Merck Millipore water system (WP612250). Commercially obtained human malignant melanoma A375 cells (Cellonex, 0012300) were cultivated in Dulbecco’s Modified Eagle’s medium (DMEM) enriched with 10% Fetal Bovine Serum (FBS), 0.1% of Amphotericin-β and Penicillin-Streptomycin. The cells were grown in T175 culture flask and maintained with 37 °C, 5% CO_2_ and 85% humidity. At around 80–90% confluency the cells were harvested and transferred into the 3.5 cm^2^ cell culture dishes at a density of 250 × 10^5^ cells/3 mL of complete media. Prior to experimentation, the cells were incubated overnight to allow cellular attachment. Commercially available 10 mg/mL of Cannabidiol (CBD) (Sigma-Aldrich, 90899-1 mL, St. Louis, MI, USA) with a molar mass of 314.46 g/mol was solubilized in 1 mL of 99.8% ethanol. Before use, CBD was further diluted to a stock concentration of 0.5 mg/mL with 19 mL of 99.8% ethanol.

### 2.2. Methods

#### 2.2.1. Synthesis and Characterization

Scheme 1A, sodium 2-((3.4-dicyanophenyl) thiol) acetate (phthalonitrile precursor) synthesis was achieved by a nucleophilic substitution of a nitro group from 4-nitrophthalonitrile (2.00 g, 0.012 mols) with a thiol group from mercapto-acetic acid sodium salt (1.62 g, 0.014 mols) in the presence of potassium carbonate (K_2_CO_3_, 1.50 g, 0.011 mols) in a 25 mL of Dry dimethylformamide (DMF) over 48 h while stirring at room temperature under an inert environment (argon). Upon completion of the reaction, the newly synthesized phthalonitrile was isolated and dried from the mixture through vacuum filtration using DMF and ethanol as a filtrate. PerkinElmer FTIR spectrometer was used to identify the functional groups of the phthalonitrile and phthalocyanine. Bruker Ascend 500 NMR spectrometer was used to obtain the NMR spectrums of both the phthalonitrile and phthalocyanine (ZnPcTS41).

Scheme 1B, zinc (II) 2(3), 9(10), 16(17), 23(24)-tetrakis-(sodium 2-mercaptoacetate) phthalocyanine was synthesized from cyclotetramerization of phthalonitrile precursor (500 mg, 2.08 mmol) in a round-bottomed flask containing 3 mL of 1-pentanol and zinc chloride (50 mg, 0.312 mmol) under an inert environment. DBU was added dropwise while stirring at 160 °C for 7 h to afford zinc (II) 2(3), 9(10), 16(17), 23(24)-tetrakis-(sodium 2-mercaptoacetate) phthalocyanine (ZnPcTS41). After the reaction, column chromatography with silica as a solid phase and DCM: 5% Methanol:10% water as a mobile phase were used to separate and isolate the desired product. The resulting structures of the ZnPcTS41 was confirmed using FTIR, NMR, MS, and elemental analysis.

**Phthalonitrile**, Yield. 82%. FTIR (KBR): 2227 cm^−1^ (C=N), 1720 cm^−1^ (C=O), 1603 cm^−1^ (Ar-C=C), 2912 cm^−1^ (C-H), 3062 cm^−1^, 3092 cm^−1^ (Ar-CH), 2581 cm^−1^ (Ar-S-C). 1H NMR (500 MHz, DMSO) δ 7.53 (dd, J = 24.6, 8.3 Hz, 2H, Ar-H), 7.28 (s, J = 8.1 Hz, 1H, Ar-H), 3.98 (s, 2H, C-H). Calc for C_10_H_5_N_2_NaO_2_S: C (50.00), H (2.10), N (11.66), S (13.35). Found C (49.82), H (2.12), N (11.52), S (13.38).

**ZnPcTS41**, Yield. 74%. Uv–Vis λ_max_/nm, DMSO. 678 (5.2). 611 (4.3). 351 (4.7). FTIR (KBR): 3352 cm^−1^(-OH), 1716 cm^−1^(C=O), 1588 cm^−1^ (Ar-C=C), 1604 cm^−1^ (C=C), 2712 cm^−1^ (Ar-S-C). 2930 cm^−1^ (Ar-CH). 1H NMR (500 MHz, D2O) δ 11.15 (s, 4H, OH), 8.44 (s, 2H, Ar-H), 8.14 (d, J = 7.3 Hz, 2H, Ar-H), 8.00–7.93 (m, 1H, Ar-H), 7.83–7.68 (m, 6H, Ar-H), 7.65–7.52 (m, 1H, Ar-H), 2.51 (s, 8H, CH). MALDI-TOF MS (m/z): Calc: 1023.23 Found 955.95 [M-3(Na^+^)]. Calc for C_40_H_20_N_8_Na_4_O_8_S_4_Zn: C (46.86), H (1.19), N (10.92), S (12.50). Found C (46.52), H (1.21), N (10.61), S ( 11.98).

#### 2.2.2. Cell Culturing and PDT

A375 cells were cultured overnight before receiving increasing concentrations of CBD (0.3 µM to 1.1 µM) and ZnPcTS41(1 µM to 8 µM). After 4 h of PS incubation, PDT-treated cells were subjected to laser irradiation (Arroyo 4210) at 636 nm for approximately 20 min to yield 10 J/cm^2^. The cells were then cultured for another 24 h in fresh media.

#### 2.2.3. Cell Viability

Cell viability was determined using the MTT (3-(4,5-dimethylthiazol-2-yl)-2,5-diphenyl tetrazolium bromide) assay (Roche, 61187100). After 24 h, post CBD and PDT/CBD treatments, the cells were washed three times with HBSS and 0.5 mg/mL of MTT labelling reagent in serum-free media was added to each plate and incubated for 4 h. After 4 h of incubation, 300 µL of solubilization buffer was added to each plate and incubated for 24 h. Subsequently, the mixture was transferred into the clear 96-well plate and analyzed. Absorbance values at 540 nm were measured using The VICTOR Nivo^®^ multimode plate reader, (PerkinElmer, HH35940080 EN). Based on the results obtained from the MTT assay, the IC50 concentration was determined by plotting a linear regression perfect fit. (The IC50 concentration of ZnPcTS4 was found to be 5.3 µM using the resultant linear equation, y = −10.974x + 108.5 and CBD was found to be 0.63 µM using the y = −35.682x + 72.631 equation.

$$\mathrm{Cell}\;\mathrm{viability}\;(\%)=((\frac{\mathrm{Absorbance}\;\mathrm{of}\;\mathrm{Sample}}{\mathrm{Absorbance}\;\mathrm{of}\;\mathrm{Control}})\times 100)$$

#### 2.2.4. Morphology

An inverted light microscope (Olympus CKX41, C5060-ADUS) with a built-in digital camera was used to observe changes in cellular architecture of cells in the control and experimental groups at a 100× magnification.

#### 2.2.5. Uptake and PS Localization

About 2.5 × 10^5^ cells/mL of A375 cells were seeded onto a 3.5 cm^2^ diameter culture plate containing sterile microscopic coverslips incubated at 37 °C for cellular attachment. After 24 h incubation, the media was replaced with fresh prewarmed media containing 5.3 μM of ZnPcTS41 and incubated for 4 h. At 4 h post incubation, the cells were then fixed on the coverslip with 4% para-formaldehyde followed by permeabilization with a solution containing 0.5% Triton X-100 in 1X PBS. Thereafter, the cells were rinsed with 1X PBS thrice and received 200 μL of intracellular organelle-specific probes, Mito-Tracker (100 nM) for mitochondria and Lysotracker (65 nM) for lysosomes, which were all incubated for 30 min at 4 °C in the dark. Furthermore, the nucleus was counterstained for 5 min with 200 μL of DAPI. The cells were washed with 1X PBS, and thereafter, coverslips were mounted onto the glass slide. The fluorescence was detected using a Carl Zeiss Axio Z1 microscope with Alexa Fluor 594 (red), DAPI (blue), and FITC filters (green).

### 2.3. Combination Therapy of Cannabidiol and ZnPcTS41 Mediated PDT

The 50% inhibitory concentrations (IC_50_) obtained from CBD- and ZnPcTS41-mediated PDT dose response studies were used in combination studies. CBD was administered 4 h after PDT treatment and incubated for 24 h. Following the combination therapy, morphological analysis, cell viability, cell death analysis, and a live/dead assay were performed on the control and experimental groups.

#### 2.3.1. Cell Proliferation

The CellTiter-Glo^®^ luminescent assay (AnaTech: Promega, PRG7571) was used to determine cellular proliferation by quantifying the amount of ATP present in metabolically active cells. Briefly, cells were washed thrice with HBSS and detached with Tryple™Select. The cells were then resuspended in 500 HBSS a 100 µL of the cellular suspension was added to an equal volume of the CellTiter-Glo^®^ Reagent (Promega) in an opaque-walled 96 multi-well plate. The VICTOR Nivo^®^ multimode plate reader (PerkinElmer, HH35940080 EN, Waltham, MA, USA) was used to measure the luminescent signal of the control and experimental groups.

#### 2.3.2. Cell Death Pathways

Annexin V-FITC/PI (BD Pharmingen™) (556570) flow cytometry analysis was used to detect the cell death mechanisms in control and experimental groups. To identify and quantify the number of cells undergoing apoptosis or necrosis, the Annexin V-FITC-PI kit was utilized in accordance with the manufacturer’s instructions. Briefly, cells were trypsinised from the 3.5 cm^2^ culture dishes with Tryple™ Select and washed thrice with ice cold 1X PBS. Thereafter, cells were resuspended in 200 μL of 1X binding buffer. A total of 100 µL of each cellular suspension was dispensed into flow cytometry tubes followed by addition of 5 µL of Annexin V-FITC solution and 5 µL of Reconstituted Propidium Iodide Staining Solution to each cell suspension. The flow cytometry tubes, and their contents were gently vortexed and incubated at room temperature for 15 min in the dark. Then, 400 µL of ice-cold 1x (*v/v*) Binding Buffer was added to all the flow cytometry tubes and cell preparations were analyzed and interpreted using the Becton Dickinson (BD) Accuri^TM^ C6 flow cytometer.

### 2.4. Statistical Analyses

Biochemical assays were done in triplicate for 3 independent experiments. Statistical analysis was performed using one-way analysis of variance (ANOVA) in Sigma Plot version 14. The Dunnett test was used to determine statistical significance between the untreated A375 control group and experimental groups treated with CBD, ZnPcTS41-mediated PDT alone and in combination with CBD. Analyzed data on graphs were represented as mean values, standard error, and statistical significance (*p* < 0.05 *, *p* < 0.01 **, and *p* < 0.001 ***). 

## 3. Results

### 3.1. Synthesis and Characterizations

#### 3.1.1. Phthalocyanine

**Figure 1:**
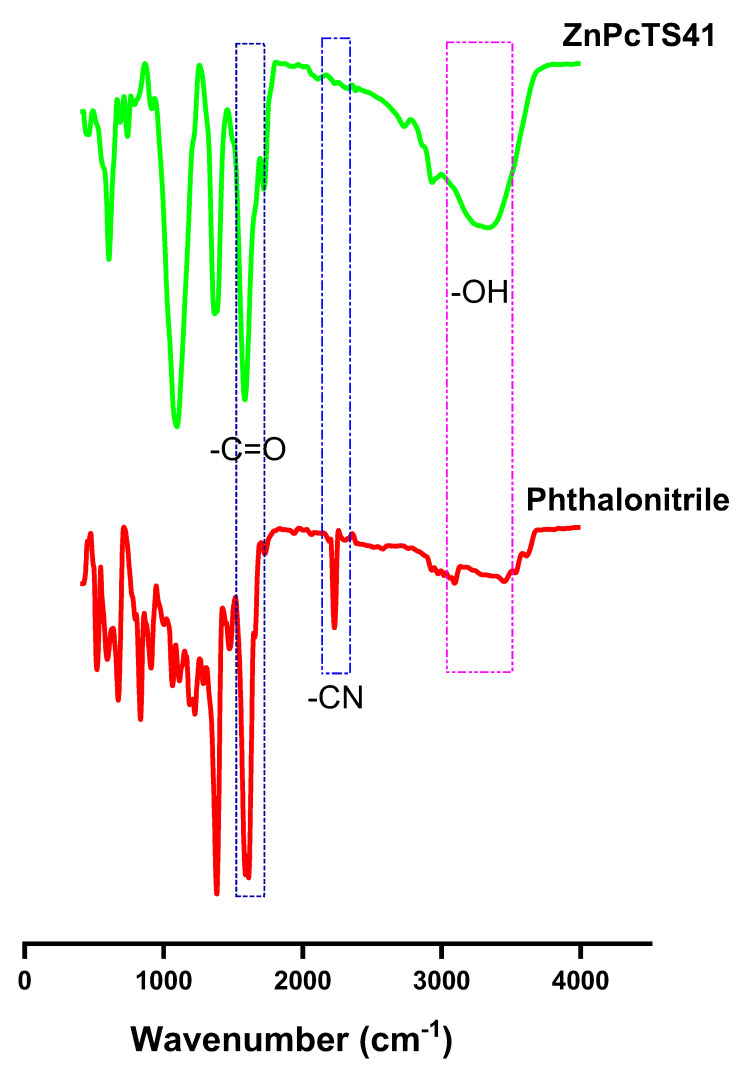
FTIR spectra of phthalonitrile precursor (sodium 2-((3.4-dicyanophenyl) thiol) acetate) and zinc (II) 2(3), 9(10), 16(17), 23(24)-tetrakis-(sodium 2-mercaptoacetate) phthalocyanines (ZnPcTS41).

The synthesis of the novel phthalonitrile and phthalocyanine derivative was performed in a step-wise process, first by the synthesis of the precursor complex (phthalonitrile) using literature methods [24,30,31] and followed by the cyclotetramerization of the phthalonitrile to form the zinc phthalocyanine [32]. Both the structure of the phthalonitrile compound and zinc phthalocyanines derivative (ZnPcTS41) were confirmed using FTIR, NMR and Elemental analysis. The infrared spectra of the phthalonitrile showed a strong stretching peak at 2227 cm^−1^ due to the nitrile (C=N) functional group of the phthalonitrile precursor, a stretching peak of a sulfide bond (Ar-S-C) at 2581 cm^−1^, a stretching carbonyl peak (C=O) at 1720 cm^−1^, an intense Aromatic-C=C stretching peak at 1603 cm^−1^, a stretching Aromatic-CH bond peaks at 3062 cm^−1^ and 3092 cm^−1^ for the aromatic ring of the phthalonitrile, thus completing the functional groups of 2-((2,3-dicyanophenyl)thiol)acetate (phthalonitrile), Figure 1. The ^1^H NMR spectra for the phthalonitrile showed a doublet of doublets peaks at 7.53 ppm and doublet peak at 7.28 ppm which integrated to two protons and a singlet for the aromatic ring, respectively. The sodium 2-mercapoacetate substituent showed only one singlet peak at 3.98 ppm that integrated to two protons belonging to the aliphatic carbon of the substituent, hence completing the 5 protons of the phthalonitrile. Elemental analysis was also used, and it confirmed the structure of the phthalonitrile by giving values in agreement with analytically calculated values.

ZnPcTS41 complex (**1)** synthesis was achieved by cyclotetramerization of 2-((2,3-dicyanophenyl)thiol)acetate through the nitrile functional groups to form the macrocyclic structure [32], Scheme 1B. The disappearance of the nitrile (-CN) peak on the infrared spectra of ZnPcTS41 confirms the successful synthesis of the phthalocyanine complex [18,33], Figure 1. Infrared spectra of ZnPcTS41 also showed stretching peaks at 1710 cm^−1^ due to the carbonyl (C=O) carbon and a broad stretching peak at 3352 cm^−1^ for the hydroxyl (-OH) group of the carboxylic acid, Figure 1. The appearance of the hydroxyl peak occurs due to exchange of the sodium spectator ion with the hydroxyl’s groups during synthesis and purifications of the complex. The aromatic ring of the ZnPcTS41 was identified by the stretching peaks at 1588 cm^−1^ (Ar-C=C), 1604 cm^−1^ (C=C) and 2930 cm ^−1^ (Ar-CH). The sulfide bond (Ar-S-C) showed a peak at 2712 cm^−1^ to complete the functional groups that make up the structure of ZnPcTS41. 1H NMR spectra of ZnPcTS41 was obtained in water due to poor peaks in DMSO, illustrated in Figure S1. The aromatic rings of the ZnPcTS41 showed a singlet peak at 8.44 ppm that integrated to two protons, a doublet at 8.14 ppm that integrated to two protons, a multiplet at 8.00–7.93 ppm with one proton integration, a multiplet at 7.83–7.68 ppm that integrated to six protons and another multiplet at 7.65–7.52 ppm that integrated to one proton to complete the cyclic structure of the ZnPcTS41. The additional substituents of the sodium 2-mercaptoacetate gave a singlet peak at 2.51 ppm which integrated to eight protons, completing the structure of ZnPcTS41. MALDI-TOF mass spectrums of the ZnPcTS41 gave a peak with atomic mass unit of less three sodium ions, indicating the dissociation that occurs during ionizations, Figure S2. Elemental analysis of ZnPcTS41 gave values in agreement with analytically calculated values.

#### 3.1.2. Ground State Electronic Absorption Spectra of ZnPcTS41

**Figure 2:**
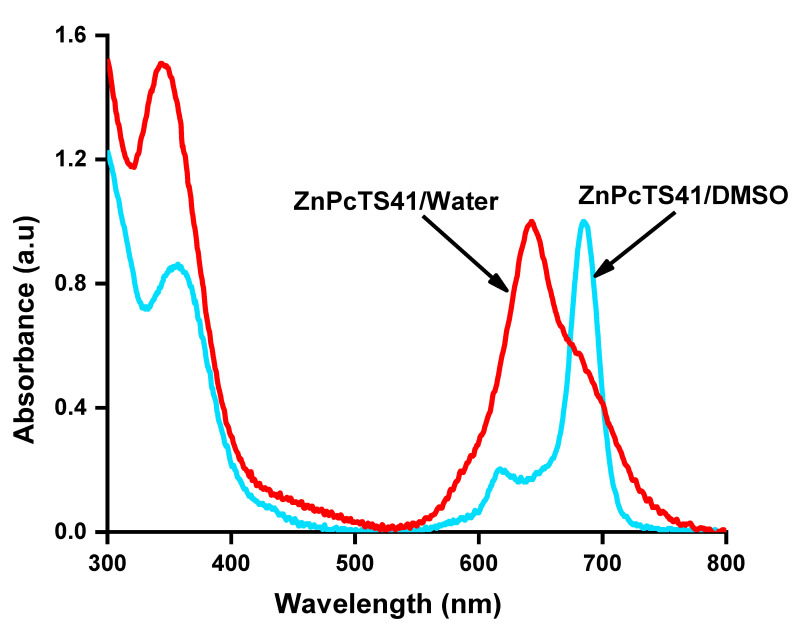
Normalized electronic absorption spectra of the ZnPcTS41 in DMSO and water.

Ground state electronic absorption spectra of ZnPcTS41 in water and DMSO is shown in Figure 2. ZnPcTS41 absorption spectra gave a typical absorption spectrum of phthalocyanine complexes in DMSO, with a Q-band, a vibronic band, and a B-band [34,35]. The Q-band maxima of the ZnPcTS41 was observed at 685 nm in DMSO and 640 nm in water, while the B-band maxima was at 350 nm for both DMSO and water Figure 2. The red-shifting of the Q-band in DMSO is as a result of its high refractive index of the solvent [36]. The electronic absorption behavior of the ZnPcTS41 plays an important role during PDT treatment as the phthalocyanine will be able to absorb light at wavelength (~640 nm) closer to the absorption in water and undergo photochemical pathways to generate cytotoxic singlet oxygen that is responsible to cancer cell destructions [5,37].

### 3.2. Dose Response Studies and IC50 Determination

#### 3.2.1. Morphological Analysis

Morphological changes of control and experimental groups of A375 cells are displayed in Figures S3 and S4. Untreated cells exhibited no abnormal changes in their morphology and maintained the normal features of melanoma cancer cells. In relation to Figure S3, treatment with varying doses of ZnPcTS41 (1, 2, 4, 6, and 8 µM) demonstrated no significant changes in cellular morphology when compared to cell only control. Similar observations were made in cells that received laser irradiation only. However, irradiated cells treated with 6–8 µM changed from their original appearance, became irregular and some round off, detached from the culture plate and appeared as free-floating cell. In Figure S4, no significant changes in cellular morphology were observed in cells treated with ethanol when compared to the control cells. This indicated that the ethanol that CBD was solubilized in had no effect on cellular viability. However, cells treated with varying doses of CBD (0.3 µM to 1.1 µM) displayed significant dose-dependent morphological changes (roundup and free-floating non-viable cells) and a decrease in cell number, inferring an increase in antiproliferative activity of CBD.

#### 3.2.2. MTT Assay (Cell Viability)

**Figure 3:**
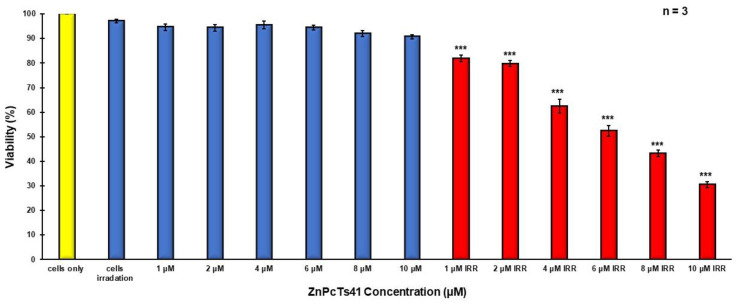
Dose responses and investigation of IC50 of ZnPcTS41 (1–8 µM) on skin cancer cells (*** *p* < 0.001).

**Figure 4:**
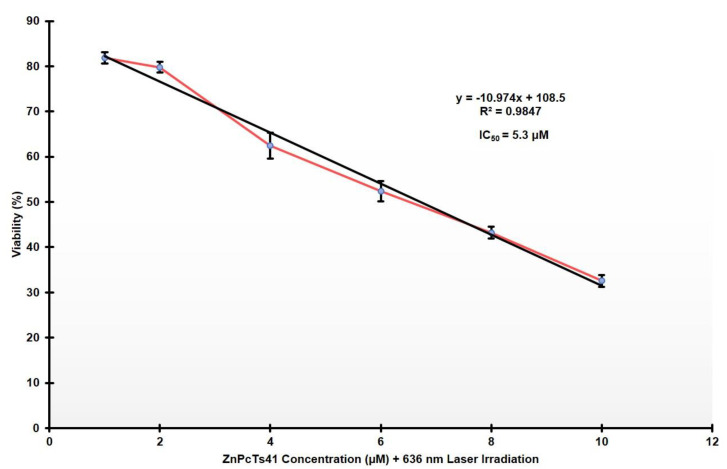
MTT assay illustrating the IC50 calculation for ZnPcTS41: y = −10.974x + 108.5.

**Figure 5:**
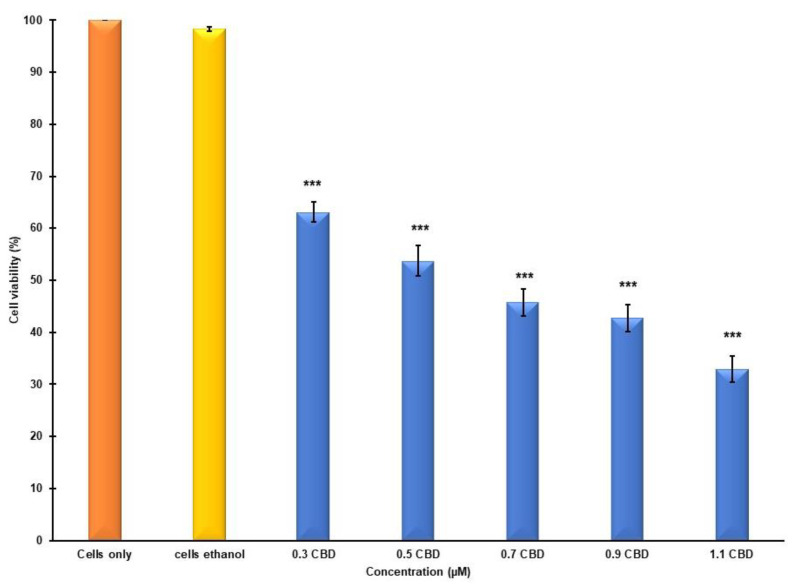
Dose responses and investigation of IC50 of CBD (0.3–1.1 µM) on skin cancer cells (*** *p* < 0.001).

**Figure 6:**
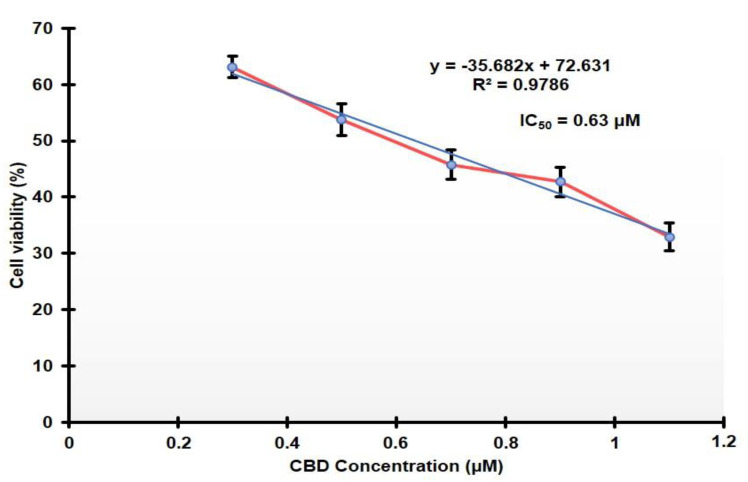
MTT assay IC50 Calculation for CBD: y = -35. 682x + 72.631.

No significant changes in cell viability were observed in cells treated with either ZnPcTS41 or laser irradiation alone when compared to the untreated control cells (Figure 3). However, a significant dose-dependent decrease in percentage viability was observed in PDT-treated cells, Figure 3 and Figure 4. In Figure 5, cell treated with ethanol alone retained a high percentage viability and demonstrated no significant changes in cellular viability when compared to untreated control cells. However, a significant decrease in cellular viability was noted in cells treated with varying doses of CBD, Figure 5 and Figure 6. Based on the results obtained from the MTT assay, the IC50 concentrations of ZnPcTS41 and CBD were determined by plotting a linear regression perfect fit, Figure 4 and Figure 6, respectively. The IC50 concentration of ZnPcTS41 was found to be 5.3 µM using the resultant linear equation, y = −10.974x + 108.5, and CBD was found to be 0.63 µM using the y = −35.682x + 72.631 equation.

### 3.3. Uptake and PS Localization

**Figure 7:**
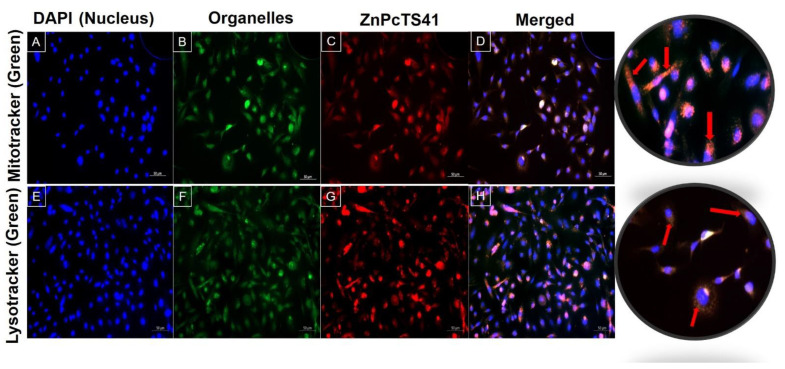
The Subcellular localization of ZnPcTS41 in A375 cells. DAPI-stained nucleus (blue fluorescence) (**A**,**E**), Mitotracker green fluorescence (**B**), Lysotracker green fluorescence (**F**), ZnPcTS41 red fluorescence (**C**,**G**), and merged images (**D**,**H**). ZnPcTS41 (red) localized on both lysosomes and mitochondria and is noted by the yellow tint between green and red (**D**,**H**) (×200 magnification).

The cellular localization site of ZnPcTS41 within A375 cells was investigated using fluorescent microscopy. The results revealed that in cells stained with mitotracker, ZnPcTS41 fluorescence depiction concurred with that of the mitochondrial marker, as indicated by an orange color, which resulted from a merged color between the green mitotracker and the red ZnPcTS41 (Figure 7D). A similar trend was observed in cells stained with the lysotracker (Figure 7H). However, the fluorescence pattern of ZnPcTS41 did not coincide with that of DAPI in any of the merged images, indicating that ZnPcTS41 did not have an affinity for the nuclei.

## 4. Combination Therapy of Cannabidiol and ZnPcTS41 Mediated PDT

### 4.1. Morphology and Viability Evaluation

**Figure 8:**
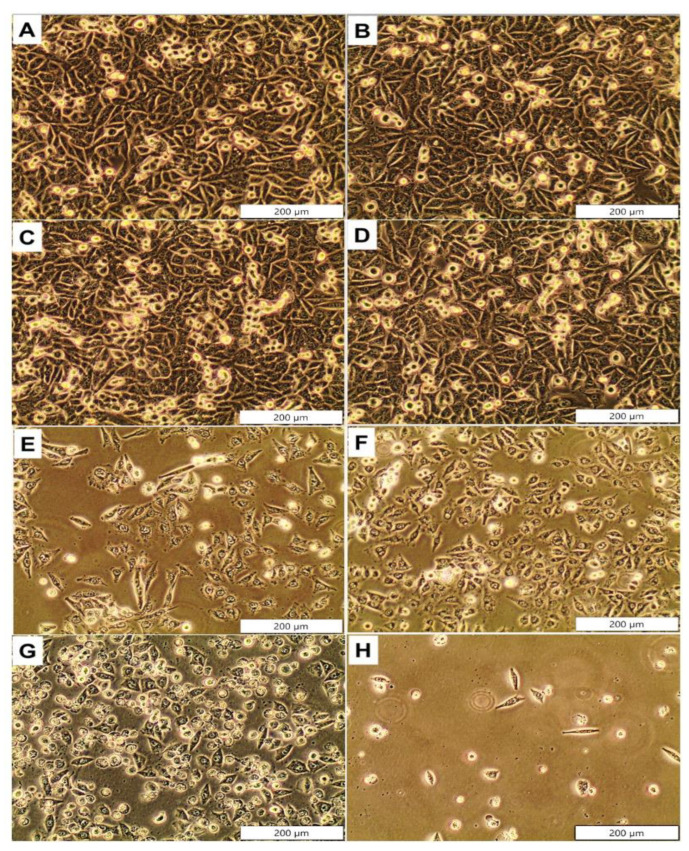
Cellular morphology of A375 cells post treatment with CBD and ZnPcTS41. untreated cells (**A**); Cells + laser irradiation (**B**); Cells + ethanol (**C**); 5.3 µM ZnPcTS4 (**D**); 0.63 µM CBD (**E**); 0.63 µM CBD + 5.3 µM ZnPcTS4 (**F**); 5.3 µM ZnPcTS4 + irradiation (**G**); 5.3 ZnPcTS4 + irradiation + 0.63 µM CBD (×100 magnification) (**H**).

**Figure 9:**
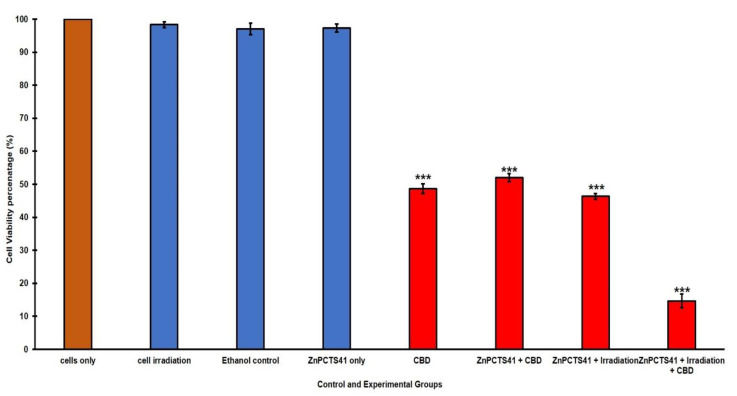
Cell viability of A375 cells 24 h post ZnPcTS41 mediated PDT treated with CBD (*** *p* < 0.001).

Untreated control cells in combination therapy did not show any morphology changes post 24 h of incubation. Similar observations were made in control cells that received ethanol, ZnPcTS41 and laser irradiation alone (Figure 8). Significant morphological changes were observed in cells treated with either CBD treatment alone or combined with ZnPcTS4, as well as in PDT-treated cells. Irradiated cells treated with 5.3 µM ZnPcTS4 and 0.63 µM CBD (Figure 8H) exhibited the most significant morphological destruction with a decrease in cell number. The cells lost their distinctive appearance, became less uniform, lost membrane integrity and attachment properties, and appeared as free-floating structures. At 24 h post combination therapy, cell viability was assessed by means of the MTT assay. No significant decrease in cell viability was observed in cells treated with either ethanol, ZnPcTS41 or laser irradiation alone as compared to untreated control cells. However, a significant decrease in cell viability was noted across all treatment groups of CBD and ZnPcTS41 IC_50_. In a similar manner, the combination experiments showed the greatest reduction in cell viability, with a staggering 15% of cells remaining viable (Figure 9).

### 4.2. Cellular Proliferation

**Figure 10:**
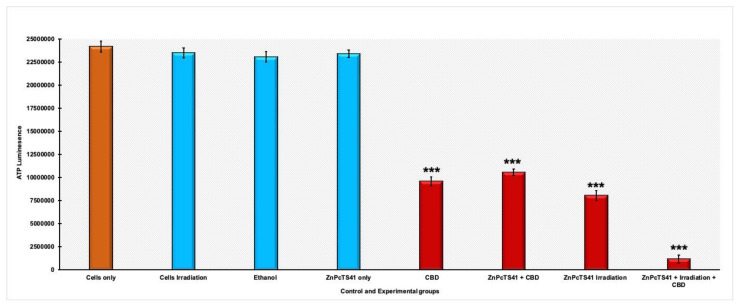
Evaluation of cellular proliferation of A375 cells treated with ZnPcTS41-mediated PDT and CBD (*** *p <* 0.001).

ATP luminescence assay was used to evaluate cellular proliferation. The untreated controls cells displayed a high luminescence signal, which was indicative of high ATP and increased proliferation rate after 24 h (Figure 10). Cells that received ZnPcTS41 alone, ethanol alone, or irradiation alone did not exhibit any significant reduction in ATP production after 24 h. There was a noticeable decrease in cell proliferation in cells treated with CBD, CBD and ZnPcTS41, ZnPcTS41 after irradiation, and ZnPcTS41-mediated PDT treated with CBD (*p* ˂ 0.001), compared to the untreated control cells.

### 4.3. Cell Death Pathways

**Figure 11:**
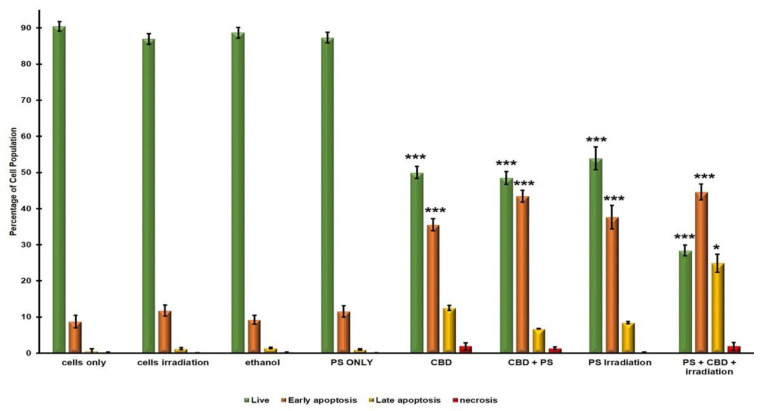
The effects of CBD and ZnPcTS41 mediated PDT on cell death of A375 cells by Annexin V-FITC/PI staining (* *p* < 0.05 and *** *p <* 0.001).

Flow cytometric analysis by means of Annexin V-FITC and PI was used to determine the mode of cell death in A375 cells following combination therapy (Figure 11). Cells treated solely with laser irradiation, ZnPcTS41 or ethanol did not display any significant changes in cell death changes when compared to the untreated cells only control. Only PDT- and CBD-treated cells showed a significant change in cell populations. The cells treated with IC50 of CBD resulted in a significant decrease in the viable cell population (50% ***) with an increase in the number of early apoptotic cells (35% ***) when compared to the untreated control. However, there was an insignificant increase in late apoptotic and necrotic cell populations. A similar trend was seen in cells treated with IC50 of CBD and ZnPcTS41 alone, which displayed an average of 48% *** viable cell population and a 43% *** early apoptotic cell population. Additionally, PDT-treated cells showed a considerable increase in early apoptotic cells (37% ***) and a significant drop in cell viability (53% ***). Combination therapy with ZnPcTS41-mediated PDT and CBD resulted in the greatest reduction in viable cell population (28% ***), with the apoptotic cell population being the most prominent (early apoptosis 44% ***, late apoptosis 25% *, and necrosis, relatively 0). 

## 5. Discussion

Photodynamic therapy (PDT) is growing in popularity as a cancer treatment due to its noninvasive nature and negligible side effects on normal tissues when compared to conventional therapies. However, resistance attributed to cancer stem-like cells hinders therapeutic developments [10]. The current study developed a novel water-soluble zinc phthalocyanine derivative (ZnPcTS41, Scheme 1) by a known base catalyzed cyclotetramerization of novel phthalonitrile precursors [32]. Typically, the cyclotetramerization reaction, Scheme 1 of the phthalonitrile complex results in a color change from a brown solution to a blue to green solution indicating the successful synthesis and metalation of the phthalocyanine complex. This is further proven by the disappearance of the sharp nitrile peak at ~2230 cm^−1^.

The complete structure and functional groups of the novel ZnPcTS41 was confirmed using FTIR (Figure 1), NMR (Figure S1), MALDI-TOF MS (Figure S2) and elemental analysis as demonstrated in the methods and results section.

Jenway Genova UV-VIS Spectrophotometer 7315 was used to analyze the ground state electronic absorption spectra of the ZnPcTS41 in DMSO and Water, Figure 2. The Q-band of the ZnPcTS41 was found to be 640 nm in water and 685 nm in DMSO. The red-shifting of the Q-band in DMSO is due to the high refractive index of the solvent. The determination of the absorption bands is vital to understanding the excitation wavelength during PDT application as the phthalocyanine derivative is used as a photosensitizer agent for the destruction of cancer cells.

ZnPcTS41 was then used as a PS agent on melanoma skin cancer cells (A375). After culture, A375 cells treated with either laser irradiated or non-irradiated ZnPcTS41 did not display any morphological alterations when compared to untreated cells (Figure S3). These clearly indicate that the PS has no dark toxicity and the 636 nm (closer to the Q-band of the ZnPcTS41 in water) diode laser at 10 J/cm^2^ does not have any photothermal effects on cancer cell killing as cells maintained their original morphology and exhibited the highest level of cell viability and proliferation. However, ZnPcTS41 in conjunction with laser irradiation led to morphological changes that appeared to be dose dependent. At a very low ZnPcTS41 doses, there were no detectable changes, but as the concentration increased, cells began to lose their distinctive morphology: they became rounded up, lost their attachment characteristic and emerged as free-floating structures in the culture media. These changes coincide with those of PDT-treated cells undergoing to cell death, as described by Naidoo et al. [38].

With reference to Figure S4, cells treated with ethanol did not display any morphological changes when compared to untreated control cells, which suggested that ethanol in which CBD was solubilized does not have any inhibitory effects on cancer cells. However, the cells treated with varying doses of CBD demonstrated significant morphological changes. These results correlate with studies conducted by Honarmand et al., which described CBD as an anticancer agent since it disrupted the cellular morphology of cancer cells [39]. Figure S3 demonstrates that the individual effects of laser irradiation or ZnPcTS41 did not trigger any significant decrease in cell viability. However, a significant decrease in cell viability percentage was observed in all PDT-treated cells. The reduction in cell viability after treatment with ZnPcS_4_ was also reported in another in vitro study [39], where the evaluated effects exhibited a dose-dependent decrease in cell viability. With reference to Figure 5, CBD treatment resulted in a dose-dependent decrease in cell viability. Similar findings were reported by Marzęda et al. [40], whereby CBD reduced melanoma cell viability in a dose-dependent manner. The same study concluded that CBD is a potent antiproliferative agent that can enhance the therapeutic efficacy of conventional anticancer therapies when administered in combination therapy [40].

PDT-treated cells coupled with CBD revealed severely significant morphological changes in relation to membrane blebbing, rounding, loss of attachment characteristics, shrinkage, as well as a significant decrease in the number of cells in the culture when compared to monotreatment of either PDT or CBD. These results correlate with the findings reported by Nkune et al., after evaluation of ZnPcS_4_ mediated PDT and CBD on cancer cells [41]. In a similar manner, the most significant decrease in cell viability was observed in PDT and CBD treated cells. These clearly indicate that the chemo preventive effects of CBD enhanced the therapeutic efficacy of ZnPcTS41-mediated PDT. Similar studies have highlighted that plant derivatives can enhance the efficiency of phthalocyanine-mediated PDT treatment of cancer [42,43]. The free radical generation following photoactivation of a PS triggers loss of viability, proliferation, and increased cytotoxicity due to the membrane disruption. These free radicals serve as units that facilitate the formation of ROS, thereby reducing cellular ATP levels [44]. The significant reduction in ATP or cellular proliferation rate in A375 cells after the treatment with ZnPcTS41 and CBD may be attributed to this reason. In addition, studies have revealed that CBD inhibits cellular proliferation in different cancer cells via modulation of the extracellular signal-regulated kinase (ERK) and ROS pathways [42,45,46,47]. The treatment with PDT in conjunction with CBD demonstrated increased potency and that may be linked to the synergistic action.

The subcellular localization of a PS is crucial as it impacts PDT’s overall outcomes [2]. The physicochemical chemical characteristics of PSs, including charge, hydrophilicity, partition coefficients, and three-dimensional architecture, are key determinants in their bioavailability, subcellular localization, and their aggregation sites within the cell, which further hinges on cellular transport mechanisms and the organelle’s membrane potential [48]. Several lines of evidence suggest that zinc phthalocyanine PSs predominately localize in the mitochondria and lysosomes of cancer cells [22,48,49,50,51]. Similar to these studies, the present study investigated the localization of ZnPcTS41 in A375 melanoma cancer cells by employing specific organelle markers (i.e., DAPI, mitochondrial and lysosomal trackers). It was noted that ZnPcTS41 preferentially localized in the mitochondria and lysosomes (Figure 7D,H).

Both Annexin V-FITC for apoptosis and PI necrosis were used to stain the control and experimental groups. Flow cytometric analysis reported that cells treated with laser irradiation alone, ethanol and ZnPcTS41 alone maintained a high level of viable cell population when compared to untreated control cells. However, when compared to the same population of untreated control cells, a significant decrease in viable cell population and an increase in the early apoptotic cell population were noted with CBD-, ZnPcTS41- and CBD-, and PDT-treated cells without CBD. Furthermore, changes in the late apoptotic and necrotic cell populations were noted; however, they were insignificant. These results are in line with studies performed by Robertson et al. which also reported that photoactivated ZnPcSmix administered to A375 cells significantly decreased viable cell population, as well as significantly increased early apoptotic cell population [51]. Furthermore, these results concurred with findings by Aviello et al., which evidenced that CBD inhibited cellular growth via induction of apoptotic cell death pathways [46]. Nevertheless, Noguchi et al. have indicated that cells undergoing early apoptosis of cell death pathway tend to be autophagic and so have the ability to rejuvenate [52]. Therefore, this early mode of apoptotic cell death is not ideal for PDT cancer treatments because cells can regenerate and cause cancer recurrence.

The most favorably significant results were attained by ZnPcTS41 mediated PDT in conjunction with CBD, which resulted in a significant decrease in the viable cell population and a significant increase in early and late apoptotic cells. Late apoptosis and necrosis are the most ideal modes of cell death in cancer therapy since cells cannot recover [52]. This significant killing of A375 cell in combination therapy can be attributed to the addition of CBD. These results are corroborated by Kenyon et al. who highlighted that CBD administration in combination with other cancer treatment therapies promotes disruption of intracellular signaling pathways like P13K/AKT/mTOR and ERK, which improves the combinative treatment cell death outcomes [53].

This study revealed the inhibitory effects of the novel ZnPcTS41-mediated PDT and CBD in A375 cells when administered in monotherapy and their enhanced antiproliferative effects when combined. The findings from this study suggest that combining anticancer therapies can increase the susceptibility of cancer cells to the treatments. Combination therapies can further lessen dose dependence by using lower doses of commercially synthesized PSs, which may potentially mitigate unwanted side effects of PDT. It was noted that apoptosis was the most predominant mode of cell death induction in different experimental groups. The overall findings from this study suggest that enhancing PDT with anticancer agents such as CBD can eliminate resistant melanoma cells, which could in turn inhibit the secondary systematic spread of cancer.

## 6. Conclusions

This study reported on the successful synthesis and characterization of a novel water-soluble ZnPc derivative (ZnPcTS41). ZnPcTS41 gave a Q-band peak at 678 nm in DMSO and 640 nm in water. The complex was used as a PS agent in PDT of human metastatic melanoma (A375), which was enhanced with CBD. The study found that ZnPcTS41 achieved an IC50 of 5.3 µM while CBD achieved an IC50 of 0.63 µM on A375 cells. Additionally, ZnPcTS41 was found to localize within the lysosomes and mitochondria of the cancer cells which facilitated cytotoxic singlet oxygen to be effective in cancer killing. The photosensitizing ability of ZnPcTS41 resulted in apoptotic cell death of A375 cells, which improved significantly when combined with CBD. The results demonstrate that combined therapy of PDT using PS agents such as ZnPcTS41, followed by anticancer agents can potentially be fitting for complete eradication of recurrence cancer cells.

## Data Availability

Not applicable.

## Supplementary Materials

The following supporting information can be downloaded at: https://www.mdpi.com/article/10.3390/pharmaceutics14112418/s1, Figures S1: NMR spectra of ZnPcTS4; Figure S2: MALDI-TOF spectrum of ZnPcTS41; Figure S3: Cellular morphology of A375 after treatment with ZnPcTS41; Figure S4: Morphological analysis of A375 after treatment with CBD.
