# Supplementary material for: Photodynamic Therapy Efficacy of Novel Zinc Phthalocyanine Tetra Sodium 2-Mercaptoacetate Combined with Cannabidiol on Metastatic Melanoma
Source: Pharmaceutics. 2022 Nov 9;14(11):2418. doi: 10.3390/pharmaceutics14112418 (PMC9695911; doi:10.3390/pharmaceutics14112418)
Supplement: Supplementary file 1 [file pharmaceutics-14-02418-s001.zip › pharmaceutics-1980385-supplementary.pdf]

Zinc phthalocyanine tetra sodium 2-mercaptoacetate enhanced with cannabidiol in photodynamic therapy of skin cancer.

Nkune Williams Nkune[1], Gauta Gold Matlou[1] and Heidi Abrahamse[1]*

[1]Laser Research Centre, Faculty of health sciences, University of Johannesburg, P.O Box 17011, Johannesburg 2028, South Africa.

*Correspondence: habrahamse@uj.ac.za.

Supplementary information.

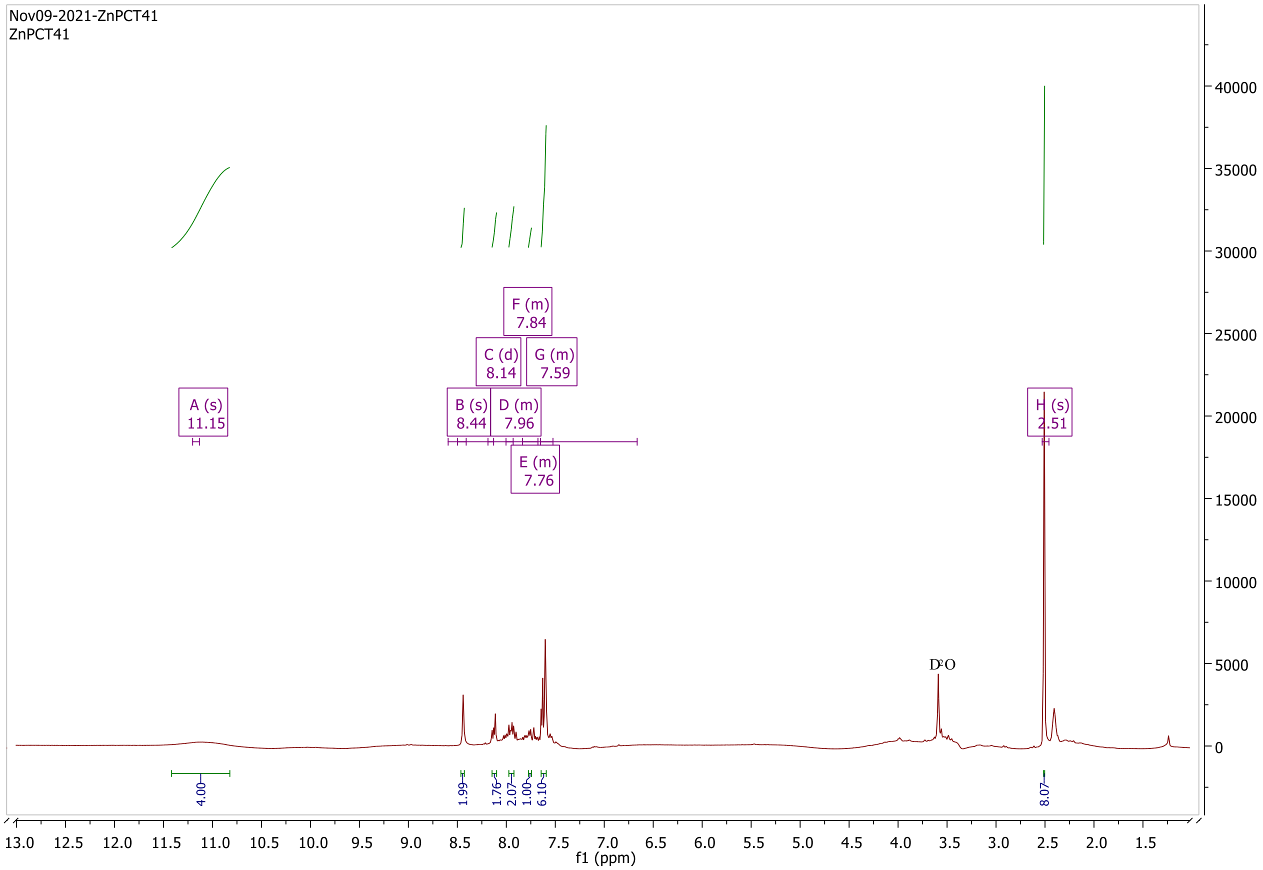

Figure S1. NMR spectra of ZnPcTS41 in D_2O

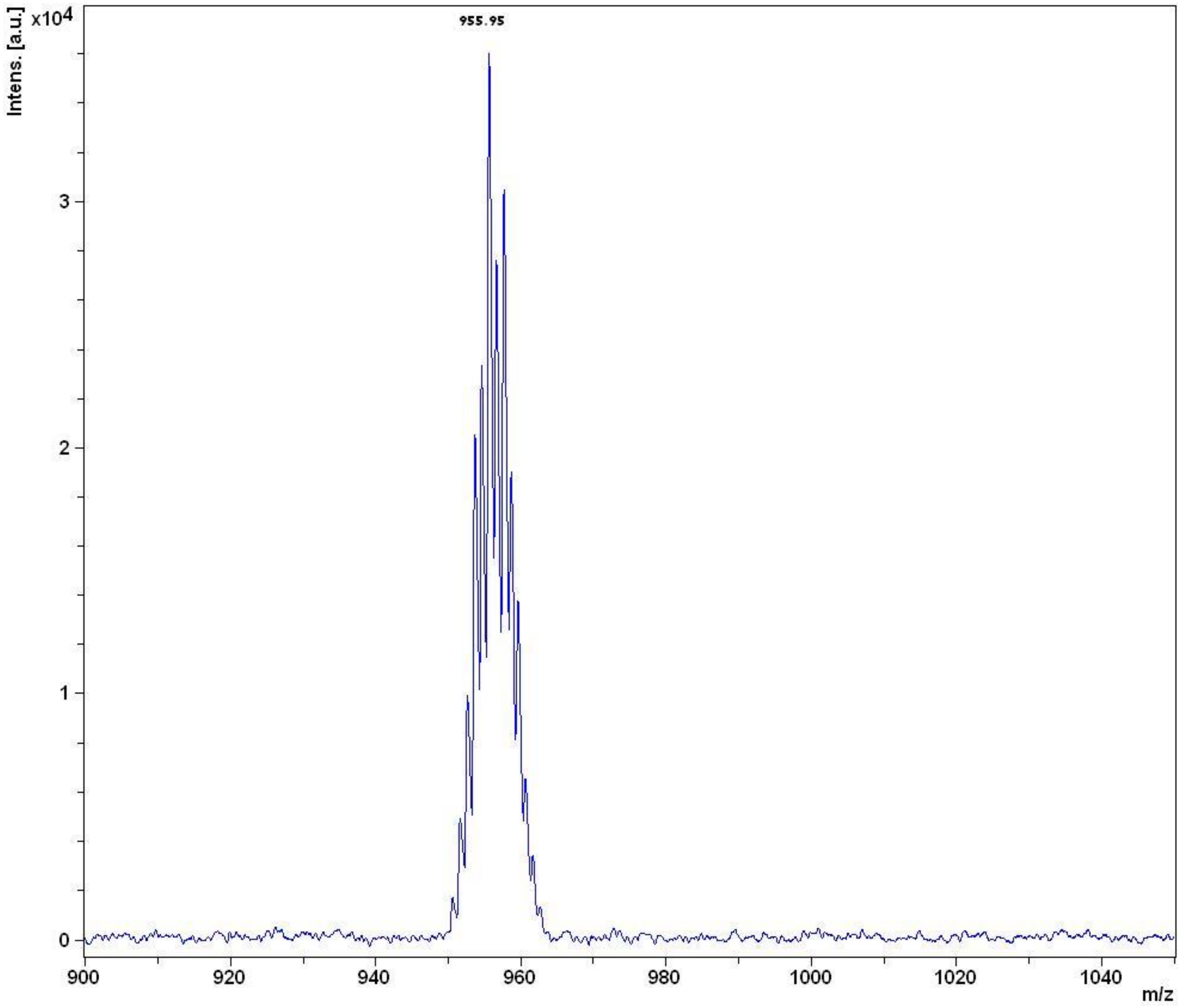

Figure S2. MALDI-TOF mass spectrum of the ZnPcTS41 using α-cyano-hydroxycinnamic acid matrix.

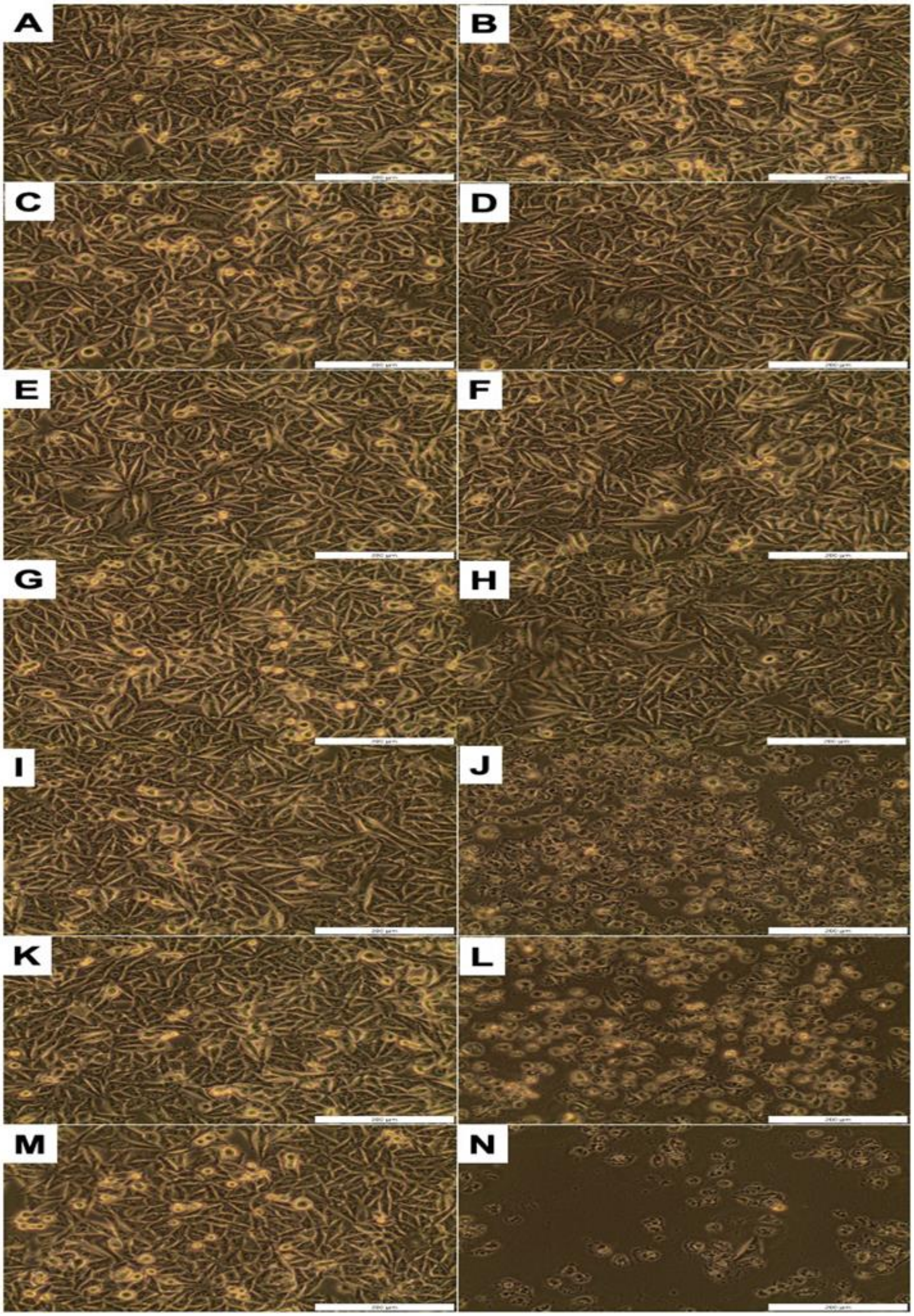

Figure S3: Cellular morphology of A375 post-treatment with ZnPcTS41 (X100 magnification). Untreated control cells (A); Cells only (B); Cells + Irradiation (C); 1 µm (D); 1 µm + Irradiation (E); 2 µm CBD (F); 2 µm + Irradiation (G); 2 µm (H); 2 µm + Irradiation (I); 4 µm (J); 4 µm + Irradiation (K); 6 µm (L); 6 µm + Irradiation (M); 8 µm (N); 8 µm + Irradiation.

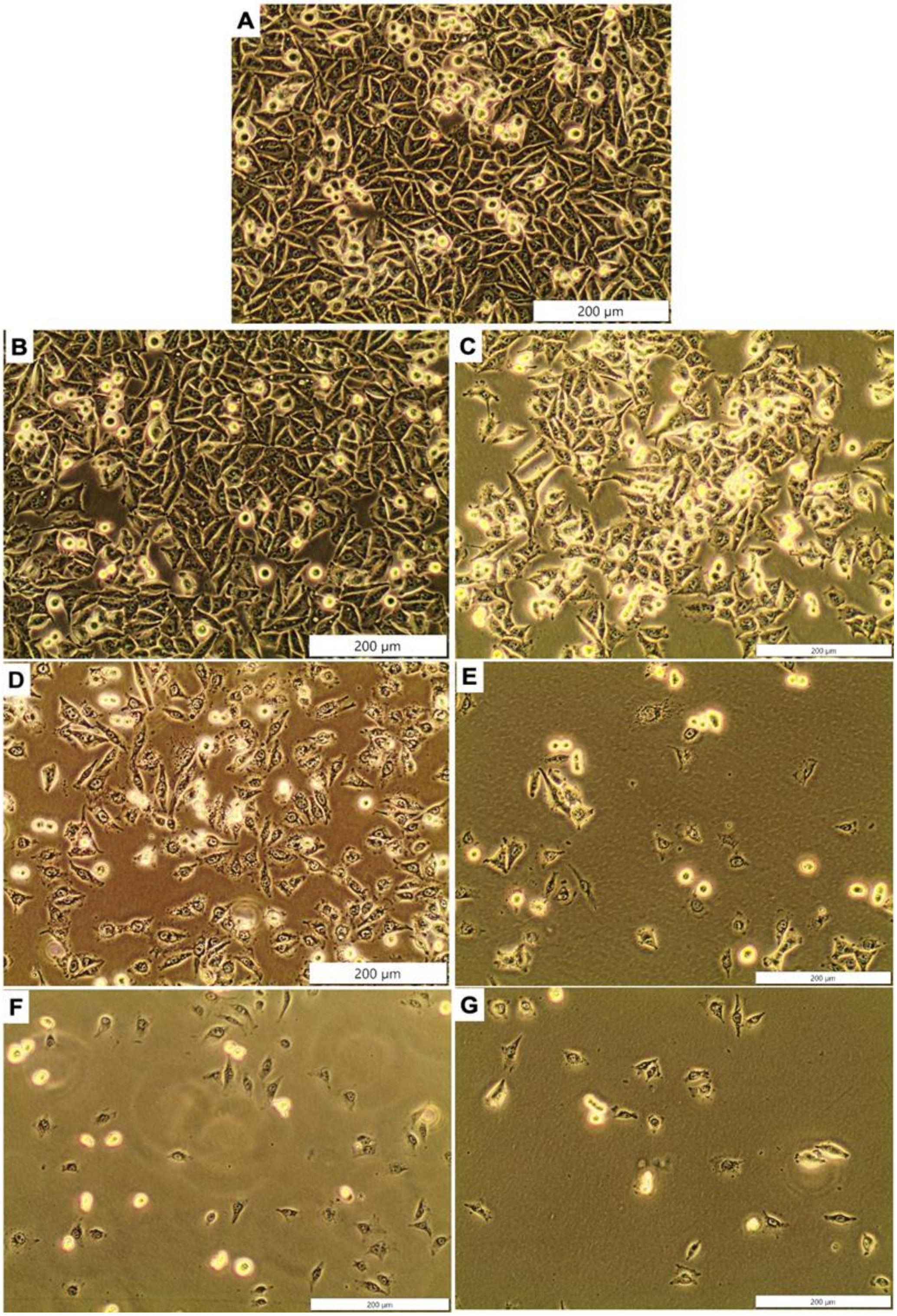

Figure S4: Morphological analysis of A375 24 h post CBD treatment (X100 magnification). untreated cells (A); Cells + ethanol (B); 0.3 µm (C); 0.5 µm CBD (D); 0.7 µm CBD (E); 0.9 µm CBD (F); 1.1 µm CBD (X100 magnification).
